# Elevated Neuropeptide Y in Endothelial Dysfunction Promotes Macrophage Infiltration and Smooth Muscle Foam Cell Formation

**DOI:** 10.3389/fimmu.2019.01701

**Published:** 2019-07-18

**Authors:** Bongkun Choi, Min-Kyung Shin, Eun-Young Kim, Ji-Eun Park, Halim Lee, Seong Who Kim, Jae-Kwan Song, Eun-Ju Chang

**Affiliations:** ^1^Department of Biomedical Sciences, Asan Medical Center, University of Ulsan College of Medicine, Seoul, South Korea; ^2^Stem Cell Immunomodulation Research Center, Asan Medical Center, University of Ulsan College of Medicine, Seoul, South Korea; ^3^Department of Biochemistry and Molecular Biology, Asan Medical Center, University of Ulsan College of Medicine, Seoul, South Korea; ^4^Division of Cardiology, Asan Medical Center, Research Institute for Valvular Heart Disease University of Ulsan College of Medicine, Seoul, South Korea

**Keywords:** neuropeptide Y (NPY), pentraxin 3 (PTX3), smooth muscle foam cell, macrophage, lipid, endothelial nitric oxide synthase (eNOS)

## Abstract

Endothelial dysfunction has been linked to vascular inflammation and foam cell formation but the underlying mechanisms still remain unclear. We sought to define the factors inducing inflammation and smooth muscle foam cell formation under endothelial dysfunction using endothelial nitric oxide synthase (eNOS)-deficient mice. Vascular smooth muscle cells (VSMCs) from eNOS-deficient mice displayed increased expression of macrophage-related genes and elevated lipid uptake. Neuropeptide Y (NPY) was upregulated in the aorta from the eNOS-deficient mice and promoted macrophage chemotaxis toward VSMCs while enhancing the activity of matrix metalloproteinase-3. Notably, NPY induced lipid uptake in VSMCs, facilitating smooth muscle foam cell formation, in association with enhanced expression of genes related to modified low-density lipoprotein uptake and macrophages. NPY was augmented by inflammatory pentraxin 3 (PTX3) in VSMCs. PTX3 enhanced macrophage migratory capacity through the NPY/neuropeptide Y receptor axis and this effect was attenuated by pharmacological inhibition with a receptor-specific antagonist. These observations suggest that endothelial dysfunction leads to the elevation of NPY that amplifies vascular inflammation by increasing inflammatory cell chemotaxis and triggers smooth muscle foam cell formation.

## Introduction

Endothelial dysfunction leads to increased permeability and subendothelial retention of modified low-density lipoprotein (LDL) particles, which trigger the subsequent recruitment of monocyte/macrophages into the subendothelial intima (1) and the initiation of plaque formation in the intima of blood vessels (2). Endothelial dysfunction due to reduced nitric oxide (NO) bioavailability is an early marker for atherosclerosis (3). Atherosclerosis is the result of complex interactions involving endothelial dysfunction with lipoprotein accumulation, inflammatory infiltration, foam cell formation, and smooth muscle cell alterations (2). Endothelium has vasoprotective effects, such as vasodilation, suppression of smooth muscle cell proliferation and migration, and inhibition of inflammatory responses (3). NO is synthesized in endothelial cells from the amino acid L-arginine by endothelial NO synthase (eNOS). NO prevents oxidative modification of LDL cholesterol (4), and a defect in the production or activity of NO leads to endothelial dysfunction.

The endothelium regulates vascular inflammation via the release of NO under physiological conditions (5). Thus, endothelium dysfunction exacerbates inflammatory responses, including inflammatory cell infiltration, the induction of cytokines, and matrix metalloproteinases (MMPs) (3). Monocyte-derived macrophages are the first inflammatory cells to infiltrate (6). First, macrophages are capable of engulfing modified forms of LDL particles to yield lipid-loaded foam cells, which form a core region of plaque together with lipid droplets (7, 8). Second, these cells secrete MMPs, leading to thinning of the fibrous cap and subsequent plaque rupture by degradation of the extracellular matrix (9). MMPs degrade extracellular molecules to release resident VSMCs and promote proliferation of these cells (9).

VSMCs within plaques express macrophage-related genes and surface scavenger receptors for LDL uptake, which makes VSMCs prone to convert into foam cells (10–12). These cells migrate from the media to the intima and proliferate (13). The formation of lipid-loaded foam cells derived from VSMCs has been documented *in vivo* and *in vitro* (11, 14). Half of the foam cells that are positive for smooth muscle alpha-actin appear to be of VSMC origin in human plaques (15).

Neuropeptide Y (NPY) is the most abundant peptide in the heart and brain, and is produced by various cells including sympathetic neurons, endothelial cells, and platelets (16, 17). NPY is involved in diverse biological functions including the stimulation of sympathetic nerves, immune function, regulation of food consumption, modulation of heart rate, vasoconstriction, coronary blood flow, and ventricular function (18–20). In particular, NPY participates in the inflammatory process by enhancing adherence (21), chemotaxis (21, 22), and cytokine secretion by monocytes/macrophages (23, 24). NPY is involved in innate and adaptive immune responses as well as metabolic alterations (18, 25, 26).

Presently, we report the upregulated expression of NPY in eNOS-deficient mice. On the basis of previous observations, we aimed to determine the role of NPY in inflammatory cell chemotaxis and formation of smooth muscle foam cells. We found that NPY driven by inflammatory pentraxin 3 (PTX3) affects smooth muscle foam cell formation by influencing the chemotaxis of macrophages.

## Materials and Methods

### Reagents

Tumor necrosis factor-alpha (TNF-α), interleukin (IL)-1β, IL-8, and PTX3 recombinant proteins were purchased from R&D Systems (Minneapolis, MN, USA). NPY and NPY receptor Y1 antagonist (BIBO3304) were obtained from Tocris Bioscience (Bristol, UK).

### Mice

*eNOS*^−/−^ mice were purchased from Jackson Laboratory (Bar Harbor, ME, USA). Female wild type or *eNOS*^−/−^ mice (8-week-old, *n* = 12) were fed a Western-type diet (42% of total calories from fat; 0.15% cholesterol; Harlan-Teklad) for 24 weeks. The animals were maintained at the Animal Center of Ulsan University (Seoul, Korea) with free access to food and drinking water under 12 h cycles of light and dark. The experiments involving mice were conducted according to the protocol approved by the Ethics Committee of Ulsan University and conformed to the Guide for the Care and Use of Laboratory Animals published by NIH. The application form included a statement guaranteeing strict observation of the animals' rights.

### Cell Culture

Murine VSMCs were isolated from the aorta by enzyme isolation as previously described (27). Cultures were trypsinized, followed by the addition of CD31-conjugated Dynabeads (Pharmingen) to the cell suspension and magnetic separation of endothelial cells (ECs) from VSMCs. Selectivity of recovered VSMC populations was highly specific based on detection of α-smooth muscle actin protein by FACS analysis in the VSMC cultures (Supplementary Figure 1). VSMCs at passage 2 to 10 were used for experiments. Murine VSMCs were grown in Dulbecco's modified Eagle's medium (Thermo Fisher Scientific Inc., Waltham, MA, USA) supplemented with 10% fetal bovine serum (FBS; Thermo Fisher Scientific), penicillin (100 U/ml; Invitrogen Life Technologies, Carlsbad, CA), and streptomycin sulfate (Invitrogen Life Technologies). Mouse bone marrow cells were prepared from the femur and tibia of 6-week old female mice and cultured with 30 ng/ml macrophage colony stimulation factor (M-CSF) (Peprotech, Rocky Hill, NJ, USA) for 3 days to prepare bone marrow-derived macrophages (BMMs) as previously described (28). BMMs were grown in Minimal Essential Media (Thermo Fisher Scientific) supplemented with 10% FBS and 30 ng/ml M-CSF.

### RNA Isolation, RT-PCR, and qPCR

Total RNA was extracted from cultured VSMCs and BMMs using Trizol reagent (Invitrogen Life Technologies) following the manufacturer's instructions. RevertAid First strand cDNA Synthesis kit (Thermo Fisher Scientific) was used to synthesize cDNA from RNA and PCR was performed in a T100^TM^-Thermal Cycler (Bio-Rad, Hercules, CA, USA). The PCR products were analyzed by electrophoresis in a 2% agarose gel and imaged using an ultraviolet gel imaging system (Bio-Rad). The qPCR analysis was performed in optical 96-well plates using SYBR Green PCR master mix (Roche, Penzberg, Germany) and the Light Cycler 480 Real-time PCR Detection System (Roche) according to the manufacturer's instructions. Gene expression was normalized to that of *glyceraldehyde-3-phosphate dehydrogenase* (*GAPDH*), which was used as an internal control. The relative expression of the target genes was calculated by the standard curve method using the target Ct values and the Ct value for *GAPDH*.

### ELISA

The amounts of secreted PTX3 and NPY protein in conditioned media (CM) were evaluated with a PTX3- (R&D Systems), and NPY- (MyBiosource, San Diego, CA, USA) specific sandwich ELISA system, following the manufacturer's protocol as described previously (28).

### Transwell Assay

Cell chemotaxis was assessed as described previously (28). VSMCs (8 × 10^4^) were plated in the lower chamber of a 5-μm pore size Transwell system (Costar, Corning, NY, USA). To evaluate the chemotactic potential of PTX3 or NPY, VSMCs in the lower chamber were exposed to PTX3 (200 ng/ml) or NPY (200 nM). CellTracker Green (5-chloromethylfluorescein diacetate, CMFDA; Invitrogen Life Technologies)-labeled macrophages (1 × 10^5^) were loaded to the upper compartment of each Transwell device. To examine the receptor-specific effect of NPY on chemotaxis, macrophages in the upper chamber were incubated with the NPY1 receptor (NPY1R) antagonist, BIBO3304 (200 nM), while PTX3 (500 ng/ml) or NPY (200 nM) was added to the bottom chamber seeded with VSMCs. After incubation for 6 h to allow for macrophage migration, macrophages that had not migrated were scrapped from the top of the membrane with a cotton swab, and the number of macrophages in the lower chamber was counted by fluorescence microscopy using a LSM700 microscope (Carl Zeiss, Jena, Germany).

### cDNA Microarray, Data Processing, and Analysis

Global gene expression analyses of aorta specimens from wild-type (WT) and *eNOS*^−/−^ mice were performed using the GeneChip® Mouse Gene 2.0 ST Array (Affymetrix, Santa Clara, CA, USA) as described previously (29). The complete data set is available with NCBI Gene Expression Omnibus (GEO) accession number GSE123855.

### Lesion Assessment and Immunohistochemistry (IHC)

Mice were anesthetized and perfused with PBS, pH 7.4. The hearts and aortas were dissected, embedded in OCT compound (Sakura Finetek, Torrance, CA, USA), and snap-frozen in liquid nitrogen. Serial 5 μm-thick sections were cut through the aortic sinus. IHC was carried out utilizing the EnVision® G|2 Doublestain System and rabbit/mouse (DAB+/Permanent Red) in accordance to the manufacturer's protocol (Dako, Carpinteria, CA, USA). Sections were fixed with paraformaldehyde and then permeabilized with 0.25% Triton X-100 in PBS. After being blocked with dual endogenous enzyme-blocking reagent, sections were incubated with anti-NPY antibody (Abcam, Cambridge, UK) for 30 min at room temperature and the polymer/horseradish peroxidase conjugate system was applied for detection. Corresponding rabbit sera were used as the negative controls.

### Immunoblot

CM containing equal amounts of protein from BMMs or cell lysates were resolved by SDS-PAGE and electophoretically transferred to a polyvinylidene difluoride membrane (Bio-Rad). Non-specific interactions were blocked using 5% bovine serum albumin solution in Tris–buffered saline (20 mM Tris/HCl, pH 7.6, 150 mM NaCl, and 0.1% Triton X-100) for 1 h. The membranes were then incubated with the anti-MMP-3 antibody overnight at 4°C. Membranes were then incubated with the appropriate secondary antibodies conjugated with HRP and immunoreactivity was detected by the use of an enhanced chemiluminescence detection kit (Millipore, Billerica, MA, USA).

### MMP-3 Activity Assay

MMP-3 activity was quantified using a spectrofluorometry assay with a fluorometric assay kit (BioVision, Milpitas, CA, USA) according to the manufacturer instructions.

### LDL Uptake Assay

VSMCs from WT and *eNOS*^−/−^ mice were incubated with Dil 488-labeled acetylated LDL (10 μg/ml; Invitrogen Life Technologies) for 30 min at 37°C. Cells were then fixed in 4% formaldehyde and internalized fluorescence was examined by fluorescence microscopy (Carl Zeiss, Jena, Germany). Raw fluorescence intensity images were analyzed and DiI fluorescence was quantitated with the software Zen 2010D (Zeiss MicroImaging, Carl Zeiss).

### Statistical Analyses

**Figure 1:**
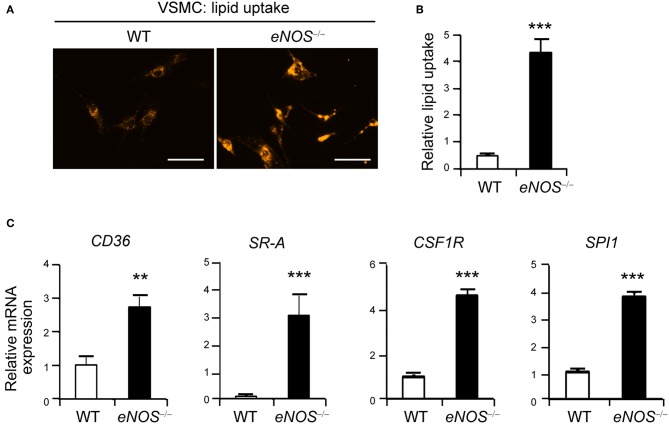
Elevated uptake of acetylated LDL in *eNOS*^−/−^ vascular smooth muscle cells (VSMCs). **(A)** Representative photographs of LDL uptake from WT and *eNOS*^−/−^ mice VSMCs. VSMCs from WT and *eNOS*^−/−^ mice were incubated with Dil 488-labeled acetylated LDL (10 μg/ml) for 30 min at 37°C. Fluorescent images show that *eNOS* null cells took up more Dil (orange) than WT. Scale bar denotes 100 μm. **(B)** The level of lipid uptake in WT and *eNOS*^−/−^ mice VSMCs was quantitated from **(A)** (means±SEM). **(C)** qPCR analysis for increased differential expression of CD36, scavenger receptor-A (SR-A), CSF1R (c-fms), and SPI1 (PU.1) in *eNOS*^−/−^ relative to WT VSMCs. GAPDH was used as an internal control (means ± SD). Data are representative of three independent experiments. ^**^p < 0.005 and ^***^p < 0.0005 compared to WT control. *p*-values were obtained using the Mann-Whitney test.

**Figure 2:**
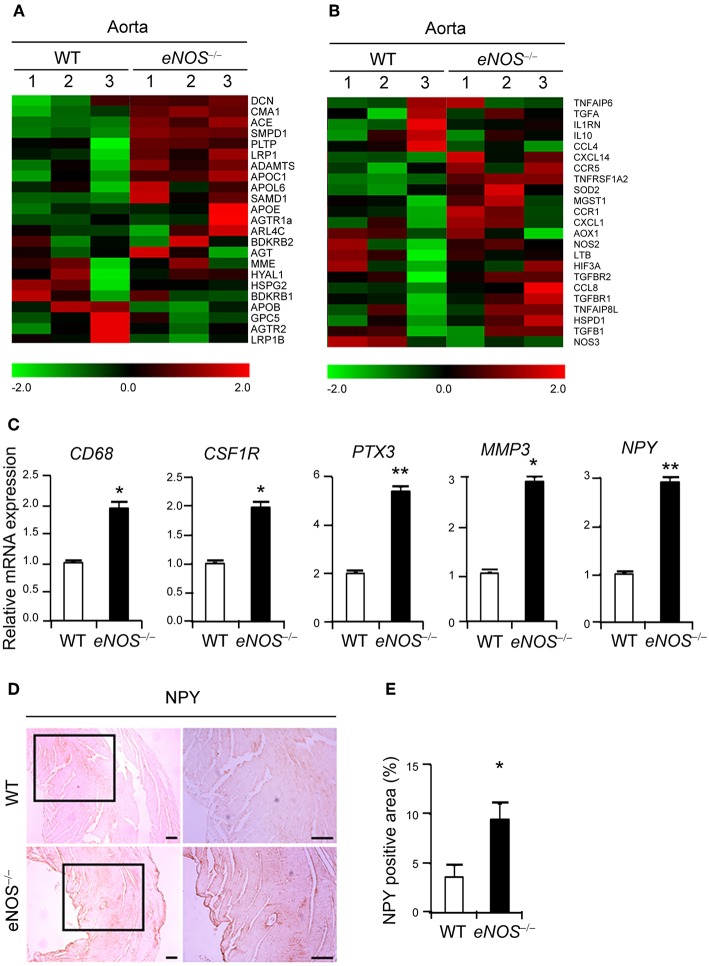
eNOS deficiency leads to increased expression of NPY. Gene expression profiles of lipid retention **(A)** and inflammation **(B)**. Transcripts that are upregulated and downregulated are shown in red and green, respectively. The columns represent the aorta samples from WT or *eNOS*^−/−^ mice. **(C)** qPCR analysis for increased differential expression of CD68, CSF1R (c-fms), PTX3, NPY, and MMP-3 in aorta tissue of *eNOS*^−/−^ mice relative to those of WT mice. GAPDH was used as an internal control. **(D)** NPY immunohistochemistry of aorta from WT and *eNOS*^−/−^ mice. Scale bar denotes 100 μm. **(E)** The percentage of NPY positive area was quantitated from **(D)**. Data are representative of more than two independent experiments. ^*^p < 0.05 and ^**^p < 0.005 compared to WT control. *p*-values were obtained using Mann-Whitney test. Data represent the mean±standard deviation.

**Figure 3:**
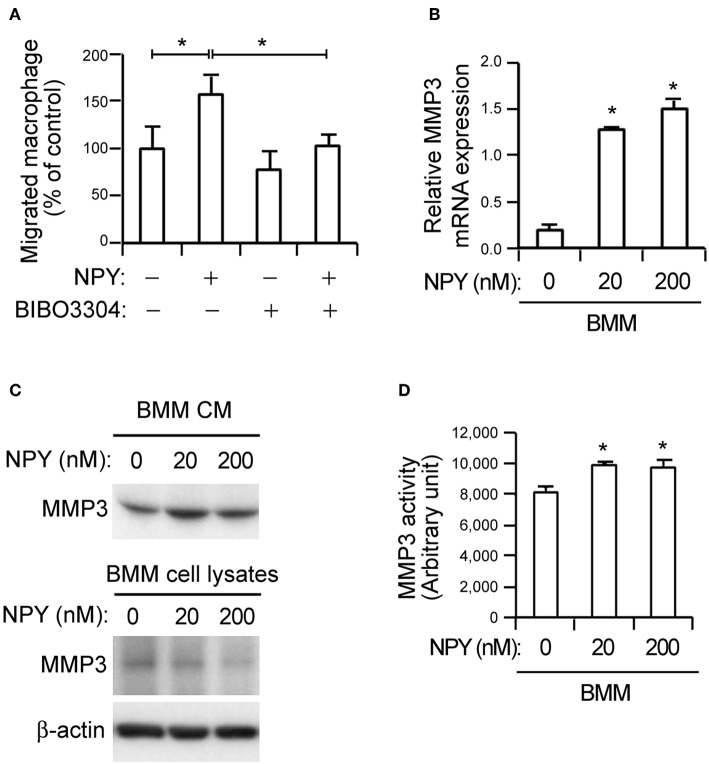
NPY promotes macrophage chemotaxis and MMP-3 activity in bone-marrow-derived macrophages (BMMs). **(A)** NPY pretreatment increases macrophage chemotaxis. Recombinant NPY proteins (200 nM) were administered to VSMCs in the lower chambers of transwell devices and neuropeptide Y receptor Y1 (NPY1R) antagonist (BIBO3304, 200 nM) was added to the upper chambers, which also contained CellTracker Green-labeled macrophages. After 6 h, the number of macrophages in the lower chamber was counted. Values represent percentage of vehicle control. **(B)** NPY upregulates the expression of MMP-3. The expression levels of MMP-3 mRNA in BMMs treated with the indicated concentration of NPY for 24 h were quantified using qPCR. Data are presented as the fold-change of the mean vehicle control value. **(C)** BMMs were incubated with indicated concentrations of NPY for 24 h and the MMP-3 proteins secreted into CM and cell lysates were detected using immunoblot assay. β-actin was included as a control. **(D)** NPY promotes MMP-3 activity in BMMs. The MMP activity in BMMs treated with the indicated concentration of NPY for 24 h were quantified using MMP-3 activity assay kit. Data are representative of three independent experiments. ^*^p < 0.05 compared to vehicle control. *p*-values were obtained using Kruskal-Wallis tests. Data represent the mean±standard error of the mean.

**Figure 4:**
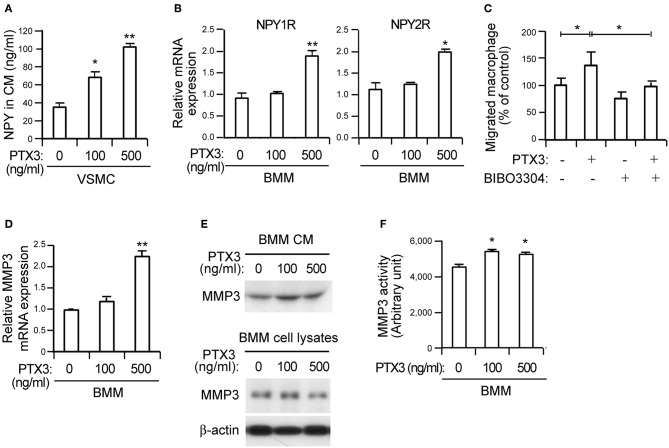
PTX3 potentiates macrophage infiltration by inducing NPY signaling and promotes MMP-3 activity in BMMs. **(A)** The protein levels of NPY in CM from VSMCs treated with the indicated concentrations of PTX3 for 48 h were measured with ELISA. **(B)** qPCR analysis for increased differential expression of NPY1R and NPY2R in BMMs treated with PTX3. GAPDH was used as an internal control. **(C)** VSMCs in the lower chambers of transwell devices were pretreated with or without recombinant PTX3 proteins (500 ng/ml) in the presence or absence of BIBO3304 (1 μM) for 24 h and CellTracker Green-labeled macrophages were added to the upper chamber. After 6 h, the number of macrophages in the lower chamber was counted. **(D)** PTX3 upregulates the expression of MMP-3. The expression levels of MMP-3 mRNA in BMMs treated with the indicated concentration of PTX3 for 24 h were quantified using qPCR. Data are presented as the fold-change of the mean vehicle control value. **(E)** BMMs were incubated with indicated concentrations of PTX3 for 24 h and the secreted MMP-3 protein into CM and cell lysates was detected using the immunoblot assay. **(F)** PTX3 promotes MMP-3 activity in BMMs. The MMP activity in BMMs treated with the indicated concentration of PTX3 for 24 h were quantified MMP-3 activity assay kit. Data are representative of three independent experiments. ^*^p < 0.05 and ^**^p < 0.005 compared to vehicle control. *p*-values were obtained using the Kruskal-Wallis test. Data represent the mean±standard error of the mean.

**Figure 5:**
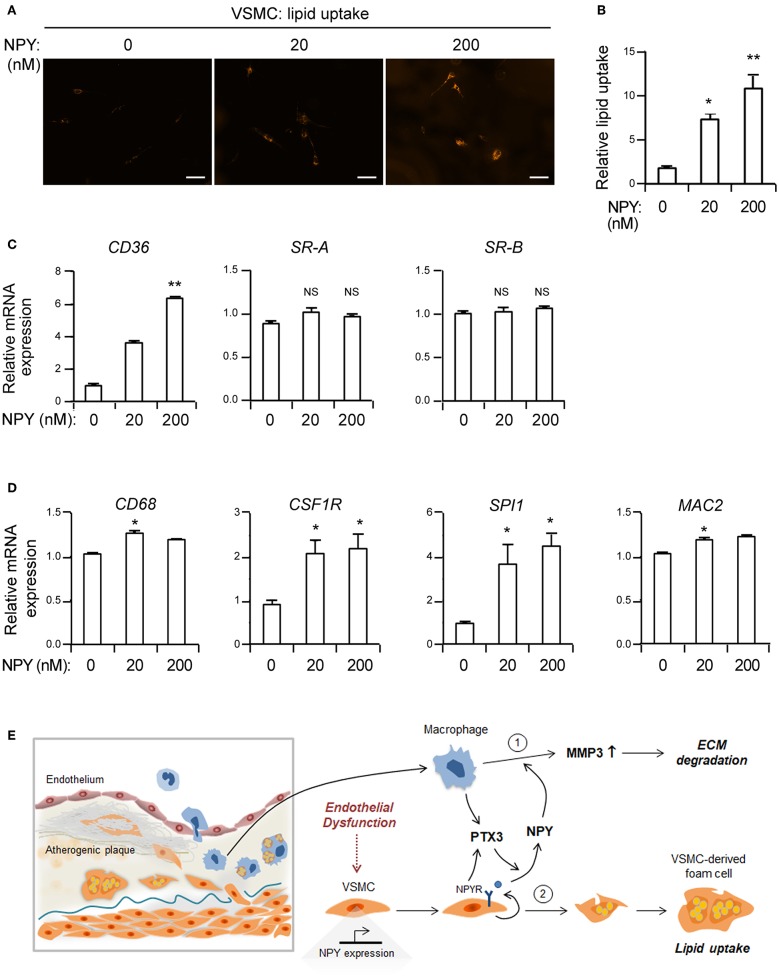
NPY elevates the uptake of acetylated LDL in VSMCs. **(A)** Representative photographs of LDL uptake from VSMCs treated with the indicated concentrations of NPY. VSMCs were incubated with Dil 488-labeled acetylated LDL (10 μg/ml) in the presence or absence of NPY for 30 min at 37°C. Smooth muscle foam cell formation after incubation with acetylated LDL was visualized by intracellular accumulation of fluorescent dye labeled lipids. Scale bar denotes 100 μm. **(B)** The level of lipid uptake in VSMCs quantitated from **(A)**. **(C)** The expression levels of CD36, SR-A and scavenger receptor-B (SR-B) mRNAs in VSMCs treated with the indicated concentration of NPY for 24 h were quantified using qPCR. Data are presented as the fold-change of the mean vehicle control value. **(D)** NPY upregulates the expression of macrophage-related genes in VSMCs. The expression levels of CD68, CSF1R, SPI1, and MAC2 mRNAs in VSMCs treated with the indicated concentration of NPY for 24 h were quantified using qPCR. Data are presented as the fold-change of the mean vehicle control value. Data are representative of three independent experiments. ^*^p < 0.05 and ^**^p < 0.005 compared to vehicle control. *p*-values were obtained using the Kruskal-Wallis test. Data represent the mean±standard error of the mean. **(E)** Proposed role of NPY in inflammation and smooth muscle foam cell formation. PTX3 augments NPY expression and NPY enhances MMP-3 activity in macrophages. Alternatively, NPY promotes lipid uptake into VSMCs leading to formation of smooth muscle foam cells.

All quantitative experiments were performed at least in triplicate. Data are presented as the mean ± standard deviation (SD) or the mean ± standard error of the mean (SEM). Mann-Whitney test (Figures 1, 2) or Kruskal-Wallis test (Figures 3–**5**) were used to compare individual groups. A *p* < 0.05 was considered statistically significant.

## Results

### Increased Lipid Uptake in *eNOS*^−/−^ VSMCs

Previous studies report that eNOS protects against atherosclerosis while eNOS deficiency leads to increased atherosclerosis in a mouse model (30–33). In addition to macrophages, VSMCs can differentiate into phagocyte-like cells and give rise to a significant number of foam cells (10, 34–36). Thus, we examined the possibility that VSMCs of *eNOS*^−/−^ mice may promote lipid uptake by visualizing the intracellular accumulation of fluorescent dye labeled lipids, Dil-labeled acetylated LDL. VSMCs isolated from *eNOS*^−/−^ mice exhibited much higher uptake of LDL compared to those of WT as evidenced by increased intracellular fluorescence-labeled lipid (Figures 1A,B). In addition, eNOS-deficient VSMCs expressed higher levels of receptors for oxidized LDL, such as CD36 (37) and scavenger receptor-A (SR-A) (38), compared to those of WT (Figure 1C). Furthermore, *eNOS*^−/−^ VSMCs expressed elevated levels of colony stimulating factor 1 receptor (CSF1R, also known as c-fms), M-CSF receptor, and SPI1 (transcriptional factor PU.1) which cause phenotypic changes of VSMCs to phagocytic cells (34) (Figure 1C), suggesting that eNOS deficiency may lead to enhanced lipid uptake by VSMCs by altering the expression of key genes implicated in the uptake of LDL.

### eNOS Deficiency Leads to Increased Expression of NPY

To define the key regulators for the uptake of LDL, we performed Affymetrix Gene Chip microarray gene expression profiling of aortic tissues isolated from WT and *eNOS*^−/−^ mice. The expressions of genes related to lipid retention (Figure 2A) and inflammation (Figure 2B) were dysregulated in the *eNOS*^−/−^ mice. Genes associated with calcification, remodeling, angiogenesis, and adhesion pathways were shown in Supplementary Figure 2. In particular, *eNOS*^−/−^ aorta tissues expressed elevated levels of CD68, a marker of macrophages (11), CSF1R, PTX3, and MMP-3 (Figure 2C). The mRNA expression of NPY, which promotes macrophage infiltration (21) was also augmented (Figure 2C). Elevated expression of NPY protein was confirmed by increased positive IHC staining in aortic tissue of *eNOS*^−/−^ mice (Figures 2D,E), suggesting that eNOS deficiency leads to increased expression of NPY.

### NPY Induces Macrophage Chemotaxis and MMP-3 Activation

Previous reports related NPY to the modulation of inflammatory reactions (21, 22). Consistent with these reports, NPY promoted macrophage chemotaxis toward VSMCs. The chemotaxis was inhibited by BIBO3304, an NPY1R antagonist (Figure 3A). MMPs derived by inflammatory signals degrade extracellular molecules to release resident VSMCs and promote proliferation of these cells, thereby accelerating atherogenesis (9). Given the observation of NPY-mediated macrophage chemotaxis (Figure 3A), we next investigated whether NPY might induce MMP expression in macrophages. qPCR analysis showed that NPY increased MMP-3 mRNA expression in BMMs (Figure 3B, *p* < 0.05). The secretion of MMP-3 protein into the CM from BMMs was concomitantly increased as detected by immunoblot assay, while intracellular MMP-3 protein expression was decreased following treatment with NPY (Figure 3C). NPY also promoted MMP-3 activity (Figure 3D, *p* < 0.05). However, NPY had no obvious effect on mRNA expression of MMP-12 and MMP-13, and there were no significant differences in the expression of these genes in WT and *eNOS*^−/−^ aorta tissue (data not shown).

### PTX3 Upregulates NPY Expression in VSMCs and Induces MMP-3 Expression in Macrophages

Endothelial dysfunction promotes sustained vascular inflammation and inflammation also promotes endothelial dysfunction (5). To identify the inflammatory factor associated with modulated NPY expression, we treated BMMs and VSMCs with various cytokines including TNF-α, IL-1β, and IL-8. These cytokines had no effect on NPY expression (data not shown). However, PTX3 exposure increased the release of NPY protein into CM in VSMCs (Figure 4A) and induced significantly higher levels of NPY1R and NPY2R in BMMs (Figure 4B). PTX3 expression is induced by TNF-α and IL-1β (28, 39). Presently, TNF-α and IL-1β significantly elevated PTX3 mRNA expression in VSMCs (Supplementary Figure 3A, *p* < 0.05). Moreover, both TNF-α and IL-1β induced PTX3 protein release into CM in VSMC cultures (Supplementary Figure 3B). These data indicate that PTX3 may induce macrophage chemotaxis toward VSMCs by upregulating the expression of NPY in VSMCs and NPYR in macrophages. The PTX3-mediated promotion of macrophage chemotaxis was abolished by the NPY1R antagonist BIBO3304 applied at 200 nM (Figure 4C, *p* < 0.05), supporting the idea that PTX3 promotes macrophage chemotaxis by activating NPYR signaling.

We next examined whether PTX3 might induce MMP expression in macrophages, similar to NPY (Figure 3). The significant upregulation of MMP-3 mRNA (Figure 4D, *p* < 0.005) was paralleled by a concomitant increase of MMP-3 protein secretion into CM from BMMs (Figure 4E) following PTX3 administration. Moreover, PTX3 promoted MMP-3 activation in similar to the effect of NPY for BMMs (Figure 4F), indicating that PTX3 may induce the expression and activation of MMP-3. Even with the PTX3-induced CD36 expression, no changes in the expression of SR-A and SR-B were observed (data not shown) and no difference in lipid uptake was noted after incubation with PTX3 in VSMCs (Supplementary Figure 4), indicating that PTX3 induced MMP-3 activation in BMMs but did not directly affect lipid uptake into VSMCs.

### NPY Induces Lipid Uptake Into VSMCs

Given the increased lipid uptake in *eNOS*^−/−^ VSMCs (Figure 1) and the elevated expression of NPY in *eNOS*^−/−^ aorta (Figure 2), we next investigated whether NPY may be involved in lipid uptake in VSMCs. NPY significantly induced uptake of LDL as compared with vehicle control as visualized by the increased intracellular location of fluorescence-labeled lipid in a dose-dependent manner (Figures 5A,B). NPY significantly induced the expression of CD36, a high-affinity scavenger receptor for uptake of oxidized LDLs into cells (37) but not SR-A and scavenger receptor-B (SR-B) (Figure 5C). Next, we evaluated the effect of NPY on the expression of macrophage-related genes in VSMCs. NPY induced mRNA expressions of both CSF1R and SPI1 (Figure 5D). In addition, the expressions of CD68 and MAC2, which are markers of macrophages (11) were enhanced by NPY treatment (Figure 5D). These results suggest that NPY may enhance lipid uptake into VSMCs and induce the formation of smooth muscle foam cells.

## Discussion

Endothelial dysfunction characterized by reduced NO bioavailability is an early marker for atherosclerosis (3) and previous reports demonstrated that eNOS protects against atherosclerosis, while eNOS deficiency leads to increased atherosclerosis in a mouse model (30, 31). We found that *eNOS*^−/−^ VSMCs manifested elevated expression of genes associated with phenotypic changes of VSMCs to phagocytic cells and induced uptake of lipid into these cells (Figure 1). Moreover, NPY expression was elevated in *eNOS*^−/−^ VSMCs (Figure 2). Pretreatment with an eNOS inhibitor attenuated the NPY induced hypotensive effects in rats (40).

Previous reports demonstrated that NPY is involved with atherosclerotic lesion formation and plaque destabilization (41–44). In particular, NPY expression is relatively elevated in unstable atherosclerotic lesions compared to stable lesions in human atherosclerotic patients and *apoE*^−/−^ mice (41). The link between NPY signaling and atherosclerotic complications is further strengthened by the identification of several single-nucleotide polymorphism in the NPY gene and a gain-of-function polymorphism associated with increased atherosclerosis in human patients (42, 43). Previous reports demonstrated the elevated NPY expression in VSMCs in atherosclerotic lesions of animal model (44), aggravation of atherosclerotic plaque by local delivery of NPY (41), and amelioration of atherosclerosis with NPY receptor antagonism (43), strengthening the notion that NPY may be implicated in atherosclerotic plaque destabilization. However, the underlying mechanisms of NPY-related atherogenesis are yet to be elucidated. NPY promotes macrophage infiltration (21) and the migration of small intestinal cells through upregulation of MMP-3 (45). Consistent with this result, NPY induced macrophage chemotaxis toward VSMCs and increased both the expression and activity of MMP-3 in BMMs (Figure 3). Furthermore, NPY provoked the phenotypic conversion of VSMCs into phagocytic cells and facilitated the accumulation of smooth muscle foam cells (Figure 5). Therefore, NPYR present in macrophages appears to be an important pathway by which NPY stimulates macrophage chemotaxis, implying that the antagonism of excessive NPYR activation could be a potential therapeutic target for the complications associated with atherosclerosis.

In this study, NPY expression was induced by PTX3 (Figure 4). PTX3, the prototype of the long pentraxin family, is an essential component of the innate immunity and involved in the regulation of inflammation and extracellular matrix construction (46). Several studies have demonstrated that the expression of PTX3 is elevated in advanced and complicated human atherosclerotic lesions, whereas no PTX3 is expressed in non-atherosclerotic internal arteries, suggesting that PTX3 may play a pivotal role in atherogenic process (47, 48). However, its roles and the precise underlying mechanism of PTX3-mediated atherogenesis yet remain uncertain. C-reactive protein (CRP), a member of short pentraxin family, is also frequently present in ruptured atherosclerotic plaque (49). CRP is mainly produced in the hepatocytes within liver in response to inflammatory cytokines, most prominently IL-6 (50), whereas expression of long pentraxin, PTX3 is stimulated by inflammatory signals at local sites of inflammation by a range of cell types (51–55) in response to inflammatory stimuli, including TNF-α, IL-1β, and lipopolysaccharide (56, 57).

It is interesting that PTX3 did not directly affect lipid uptake into VSMCs (Supplementary Figure 4). PTX3 increased NPY expression (Figure 4) and NPY induced lipid uptake into VSMCs (Figure 5). Thus, PTX3 may be indirectly involved in smooth muscle foam cell formation through NPY. Furthermore, proinflammatory cytokines such as TNF-α and IL-1β enhanced PTX3 expression in macrophages (Supplementary Figure 5). In the present study, inflammatory cytokines promoted PTX3 expression in VSMCs (Supplementary Figure 3) and macrophages (Supplementary Figure 5), PTX3 induced NPY expression in VSMCs, and PTX3 mediated the recruitment of macrophages (Figure 4). Given the prior findings of direct chemotactic effects of NPY on monocytes and macrophages (21, 22), it is likely that PTX3 promotes the macrophage chemotaxis toward VSMCs by the upregulation of NPY in VSMCs and NPYR expression in macrophages (Figure 4). These results suggest that PTX3 acts as a chemoattractant and it appears that PTX3-mediated chemotaxis occurs through NPY and NPYR activation. The infiltrating macrophages may adversely affect lesion formation and complications by providing additional TNF-α and IL-1β (58) and, in turn stimulates PTX3 expression from VSMCs. Oxidized LDL mediates its proinflammatory effects through the activation of nuclear factor-kappa B, a transcriptional factor known to be required for PTX3 expression (47, 59). These observations together with our present findings suggest that the cells present in atherosclerotic lesions may stimulate PTX3 expression in response to lipid-mediated inflammatory signals, such as TNF-α and IL-1β.

It is well-established that eNOS-deficient mice had increased blood pressure (60–63) while eNOS-overexpressing mice had lower blood pressure (64). We are currently investigating potential effect of elevated blood pressure in eNOS-deficient mice and eNOS signaling in VSMCs on atherosclerosis development. We observed increased expression of NPY in eNOS-deficient mice (Figure 2). However, further studies will be required to determine the precise mechanisms underlying the endothelial dysfunction-mediated NPY induction.

In conclusion, the present study extends our knowledge of pathological contribution of NPY/PTX3. NPY induced by PTX3 promotes lipid uptake by VSMCs and augments formation of smooth muscle foam cell. PTX3 acts as a potent chemotactic agent for macrophages by activating NPY and its respective receptor, leading to accelerated inflammation response (Figure 5E).

## Ethics Statement

*eNOS*^−^/^−^ mice were purchased from Jackson Laboratory (Bar Harbor, ME, USA). The animals were maintained at the Animal Center of Ulsan University (Seoul, Korea) with free access to food and drinking water under 12 h cycles of light and dark. The experiments involving mice were conducted according to the protocol approved by the Ethics Committee of Ulsan University and conformed to the Guide for the Care and Use of Laboratory Animals published by NIH. The application form included a statement guaranteeing strict observation of the animals' rights.

## Author Contributions

BC, M-KS, E-YK, and J-EP performed the experiments. HL, SWK, J-KS, and E-JC designed the study. BC, M-KS, and E-JC prepared the manuscript.

### Conflict of Interest Statement

The authors declare that the research was conducted in the absence of any commercial or financial relationships that could be construed as a potential conflict of interest.

## Abbreviations

BMMs: bone marrow-derived macrophages
CM: conditioned media
CMFDA: 5-chloromethylfluorescein diacetate, CellTracker Green
CRP: C-reactive protein
eNOS: endothelial nitric oxide synthase
GEO: Gene Expression Omnibus
IL-1β: interleukin-1 beta
LDL: low-density lipoprotein
MMPs: matrix metalloproteinases
NO: nitric oxide
NPY: Neuropeptide Y
NPYR: Neuropeptide Y receptor
PTX3: Pentraxin 3
qPCR: quantitative real time PCR
TNF-α: tumor necrosis factor-alpha
VSMCs: vascular smooth muscle cells
WT: wild-type.
