# Supplementary material for: Elevated Neuropeptide Y in Endothelial Dysfunction Promotes Macrophage Infiltration and Smooth Muscle Foam Cell Formation
Source: Front Immunol. 2019 Jul 18;10:1701. doi: 10.3389/fimmu.2019.01701 (PMC6657015; doi:10.3389/fimmu.2019.01701)
Supplement: Supplementary file 1 [file Data_Sheet_1.docx]

**Supplementary Information**

**Supplementary Figure Legends**


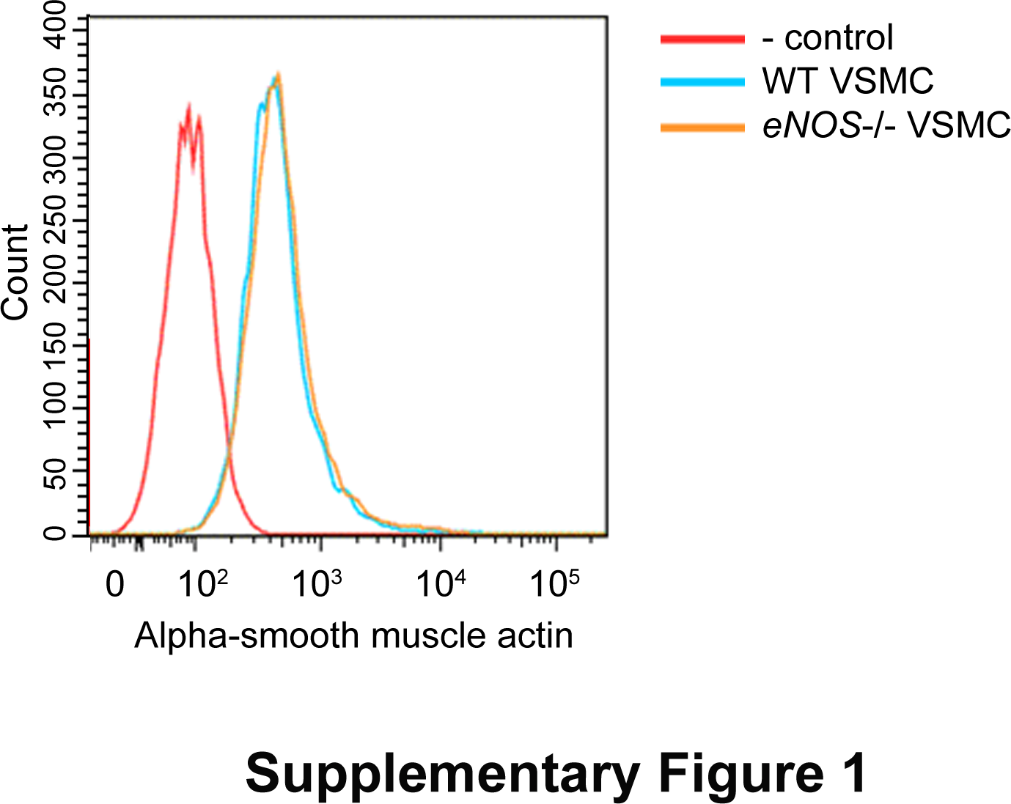


**Supplementary Figure 1.** **Representative FACS analysis of α-smooth muscle actin expression in VSMCs.** Cells isolated from aorta of WT or *eNOS*-/- mice were trypsinized, followed by the addition of CD31-conjugated Dynabeads (Pharmingen) to the cell suspension and magnetic separation of endothelial cells (ECs) from VSMCs. Selectivity of recovered VSMC populations was highly specific based on detection of α-smooth muscle actin protein by FACS analysis in the VSMC cultures.


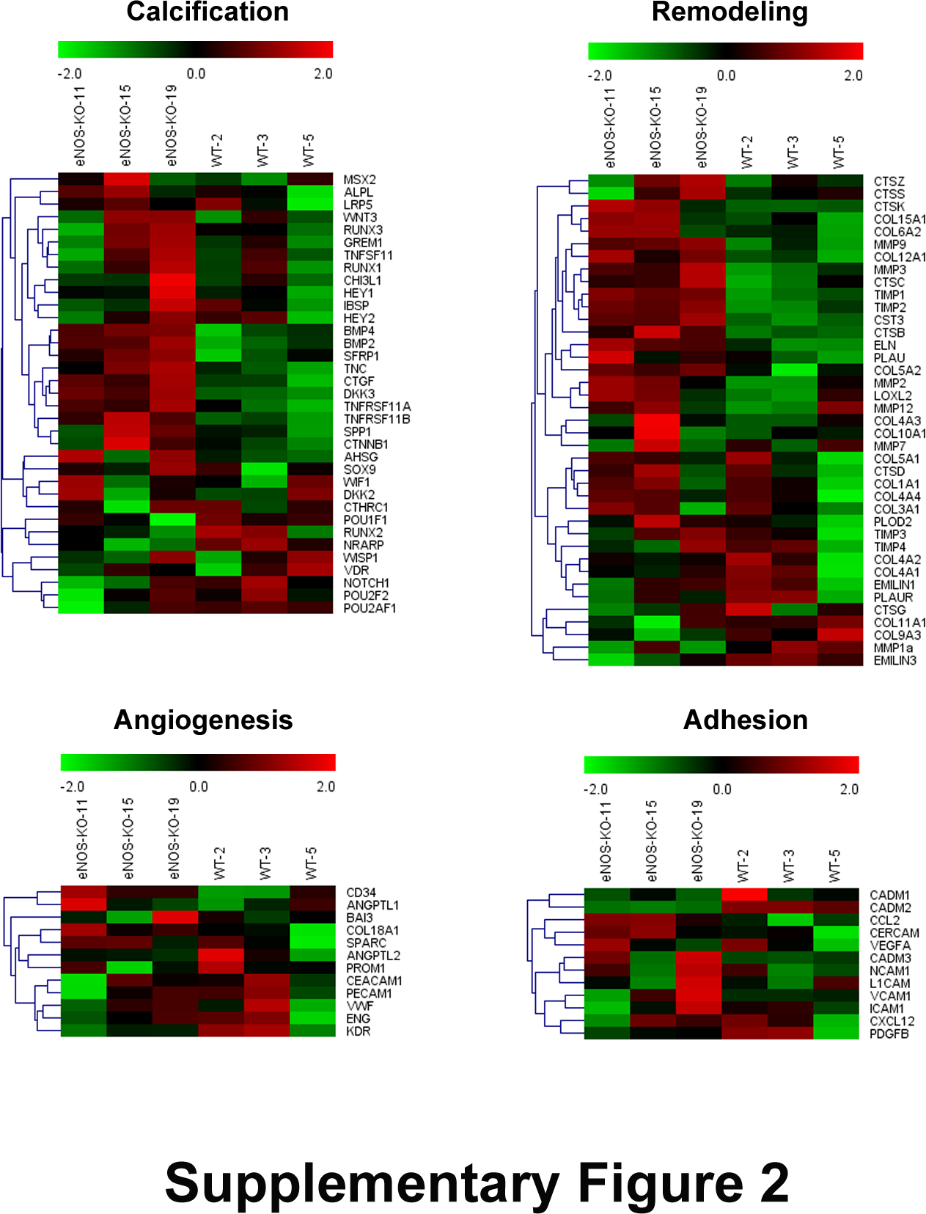


**Supplementary Figure 2.** **Altered gene expressions in aorta tissue from *eNOS*-/- mice.** Gene expression profiles of calcification, remodeling, angiogenesis, and adhesion. Transcripts that are upregulated and downregulated are shown in red and green, respectively. The columns represent the aorta samples from WT or *eNOS*-/- mice.


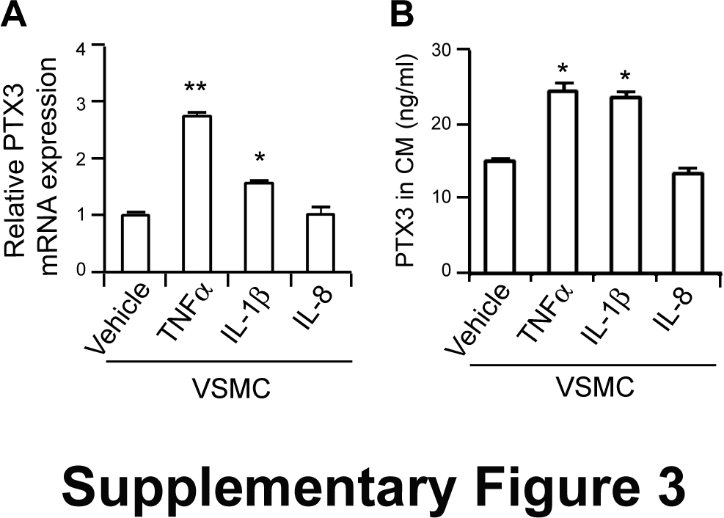


**Supplementary Figure 3. TNF-α and IL-1β induce PTX3 expression in VSMCs.** (**A**) VSMCs were treated with various cytokines, including TNF-α, IL-1β, and IL-8 (10 ng/ml) for 24 hours and the mRNA expression level of PTX3 was determined using qPCR. GAPDH was used as an internal control. Data are presented as the fold-change of the mean vehicle control value. (**B**) The protein levels of PTX3 in conditioned media (CM) from VSMCs treated with indicated cytokines for 48 hours were measured with ELISA. Values represent percentage of vehicle control. *p*-values were obtained using Kruskal-Wallis test. Data represent the mean±standard deviation.


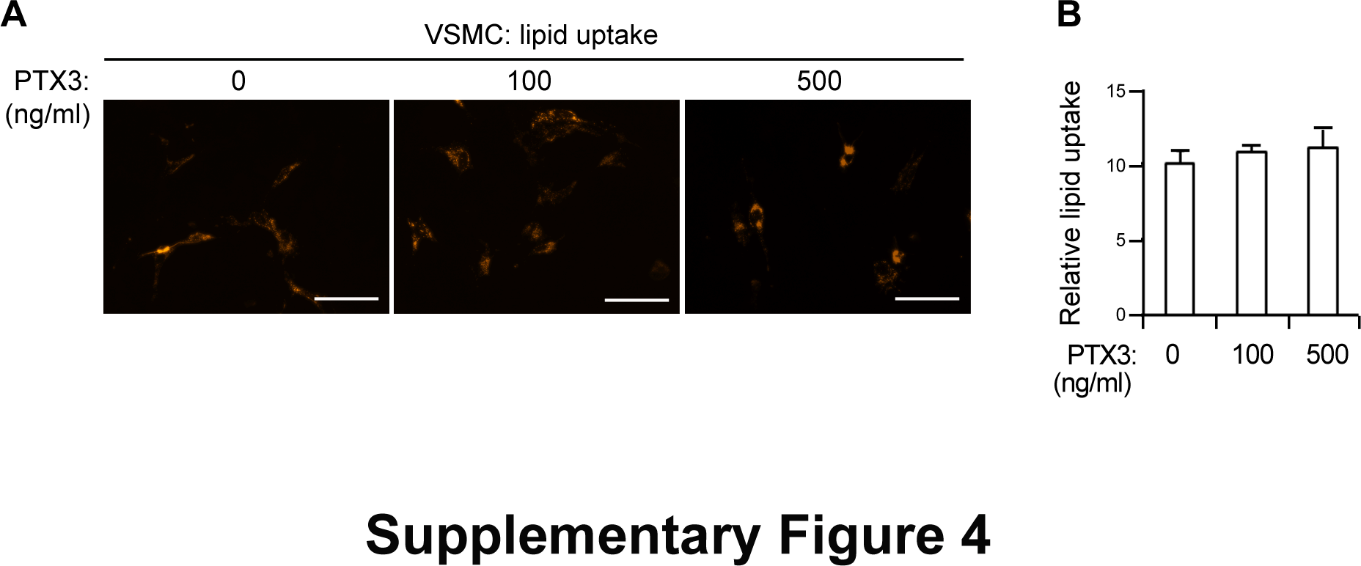


**Supplementary Figure 4. PTX3 has no effect on lipid uptake in VSMCs.** (**A**) Representative photographs of LDL uptake from VSMCs treated with the indicated concentrations of PTX3. VSMCs were incubated with Dil 488-labeled acetylated LDL (10 µg/ml) in the presence or absence of PTX3 for 30 minutes at 37°C. Scale bar denotes 100 μm. (**B**) The level of lipid uptake in VSMCs was quantitated from (**A**). *p*-values were obtained using Kruskal-Wallis test. Data represent the mean±standard deviation.


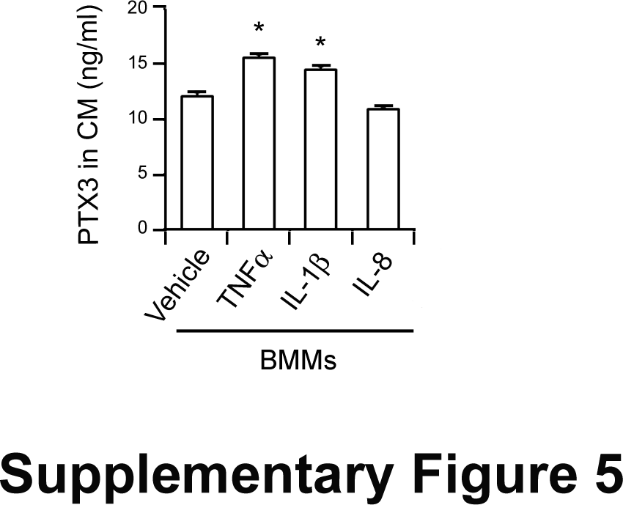


**Supplementary Figure 5. TNF-α and IL-1β induce PTX3 expression in macrophages.** Bone-marrow-derived macrophages (BMMs) were treated with various cytokines, including TNF-α, IL-1β, and IL-8 (10 ng/ml) for 48 hours and the protein levels of PTX3 in CM from BMMs were measured with ELISA. *p*-values were obtained using Kruskal-Wallis test. Data represent the mean±standard deviation.
